# Obesity and COVID-19

**DOI:** 10.3389/fendo.2020.581356

**Published:** 2020-09-30

**Authors:** Domenico Azzolino, Matteo Cesari

**Affiliations:** Department of Clinical Sciences and Community Health, University of Milan, Milan, Italy

**Keywords:** aging, inflammation, nutrition, SARS - CoV-2, body mass Index, sarcopenic obesity, ACE2 (Angiotensin Converting Enzyme-2)

The global pandemic of COVID-19 is putting a strain on the weakness of health care systems. The lack of an established treatment against COVID-19 infection and the rationing of care resulted in a dramatic scenario. Patients with COVID-19 present with very heterogeneous symptoms from asymptomatic forms to severe acute respiratory distress. Infected patients are more likely to have elevated levels of inflammatory markers, and most of those developing severe forms of the disease require mechanical ventilation. The worst-hit population comprises older people and those with multimorbidity. In particular, obesity (usually measured with body mass index, BMI) is repeatedly reported as a major risk factor for severe complications of COVID-19 (1, 2), including respiratory failure, the need for invasive mechanical ventilation, and death (3).

The role of obesity is of great relevance, especially given its high prevalence worldwide. However, it is important to bear in mind that the assessment of obesity *via* BMI is extremely arguable, especially in older people. First, it should be noted that BMI does not purely reflect adiposity because its numerator (i.e., body weight) results from the sum of both fat and fat-free mass. Second, the cut points used to categorize overweight and obesity are arbitrary and based on young and middle-aged cohorts and are, thus, inadequate for older persons (4). Third, with aging, body fat tends to accumulate in parallel with the muscle mass decline. Consequently, obesity is often underestimated in older persons, who may present an excess of adiposity within a normal/overweight body size (the so-called sarcopenic obesity) (5). Finally, ethnic difference may determine major variability in body fat distribution, in particular for what concerns ectopic and visceral fat (4). Body composition is an important aspect to consider in older persons, but it is something not easy to routinely assess even in normal times. Preventive strategies for limiting the spread of the coronavirus as well as the scarce attention frequently given to this evaluation may further accentuate this issue in the period of COVID-19. For this reason, whereas the most accurate techniques (e.g., magnetic resonance imaging, computed tomography, dual energy X-ray absorptiometry) may be difficult to implement or unfeasible during a pandemic, alternatives coming from less accurate methodologies (e.g., bioelectrical impedance analysis) and/or surrogate parameters (e.g., estimates coming from prediction equations) should be considered (6, 7).

Although evidence is still very limited, several reports are starting to indicate a role for adiposity in the COVID-19 susceptibility and severity. Just recently, Watanabe et al. (8) find that visceral fat was significantly higher in COVID-19 patients requiring ICU support—together with age, inflammation markers, and interstitial pneumonia severity. Similarly, Battisti et al. (9) report that COVID-19 severity is associated with abdominal adipose tissue distribution. In another recent study (10), the authors find a positive association between visceral adipose tissue and upper abdominal circumference with COVID-19 severity. What is more, Yang et al. (11) document that, in COVID-19 patients, visceral adiposity or high intramuscular fat deposition increases the risk for critical illness.

The contribution of obesity to the severity of COVID-19 may be explained in multiple ways. Obesity is a well-recognized risk factor for diabetes, hypertension, and cardiovascular disease, all of which are predictors of poor outcomes in COVID-19 (12). Obesity may also impair immune response to viral infections and affect diaphragm excursion (thus causing dysventilation) (3, 13). Additionally, the management of obese patients with COVID-19 might be more challenging than routine because the patient’s size may limit medical and assistive procedures.

Furthermore, obesity is characterized by abnormal secretion of adipokines and cytokines determining a low-grade systemic inflammation, which may represent the background predisposition to the most severe consequences of COVID-19 (3). At the same time, chronic low-grade inflammation represents a hallmark of aging, responsible for altered metabolism (i.e., elevation of resting energy expenditure) and increased muscle catabolism (14, 15). Inflammation could be the key factor of the muscle decline observed in older individuals, which can be further exacerbated in those with obesity (16). Obesity may also lead to increased fat infiltration of the muscle associated with a decrease in muscle strength and function, mainly due to physical inactivity of obese individuals. As a consequence, muscle decline may lead to a decrease in physical activity, which, in turn, promotes obesity with consequent increased catabolism and anabolic resistance, thus creating a vicious circle of muscle decline (17). In fact, fat mass increase usually precedes a loss of muscle mass. In other words, the increase of adiposity (especially in visceral fat) along with the low-grade chronic inflammation seen with aging could have even more detrimental effects and determine an accelerated muscle decline. Interestingly, intermuscular adipose tissue is shown to contribute to physical impairment, enhancement of insulin resistance, and increased the risk of negative health-related events (18).

Additionally, an association between the SARS-CoV-2 infection and the angiotensin-converting enzyme 2 (ACE2) receptor is widely reported, potentially playing a critical role in the pathological pathway (19–21). Interestingly, the expression of the ACE2 receptor is particularly present in adipose tissue (22, 23), possibly explaining the higher susceptibility, greater severity, and worse prognosis for COVID-19 infection in obese patients (23, 24). A controversy, however, exists about the protective or deleterious role of angiotensin-converting enzyme inhibitors (ACEIs) and angiotensin receptor blockers (ARBs) in COVID-19 (21).

In this global pandemic, the negative effects of obesity are not confined solely to the acute care setting. In fact, the need for social distancing and isolation during the lockdown may exacerbate depressive symptoms. Furthermore, self-isolation may increase the barriers to accessing healthy and fresh foods with a net shift toward convenience foods, especially in people with a poor socioeconomic status. The self-isolation period due to the lockdown may also lead to the deterioration of the circadian rhythm, resulting in a change of eating habits. Obesity has been widely related to sleep alteration and vice versa (25). In fact, sleep disorders can result in metabolic (i.e., decreased glucose tolerance and insulin sensitivity) and endocrine alterations (i.e., reduced leptin levels, high evening concentrations of cortisol, ghrelin, and increased hunger and appetite), all of which promote obesity (26). Sleep disturbances may also result from high circulating levels of pro-inflammatory cytokines, which are a hallmark of obesity (27). Additionally, high-fat and high-carbohydrate meals may alter sleep indexes (26–29) *via* the elevation of the circulating levels of glucose, insulin, leptin, cholecystokinin (CCK), peptide YY, and enterostatin (28–30).

On the other hand, it should not be neglected that obese individuals may suffer from stigma and depressive symptoms already in normal times. This may render them more likely to restrict their social contacts with detrimental consequences to their physical and psychological domains in the period of COVID-19 (31).

In conclusion, it is necessary that special attention is paid to prevent and control the COVID-19 infection in specific populations, such as obese and older persons, who are already exposed to basal inflammatory status. Inflammation, which is a hallmark of both obesity and the aging process, might have a synergistic role in promoting greater severity of COVID-19. However, as discussed, obesity measured with BMI does not necessarily reflect adiposity. Indeed, it is important to bear in mind that even though the box may look the same, the contents may be different. In this case, it would be better to think inside the box. The role of obesity in COVID-19, given the burden it poses, must no longer be ignored and may have major implications in the public health strategy.

## Author Contributions

DA contributed to conceptualizing and writing the manuscript. MC edited and revised manuscript. DA and MC approved the final version of manuscript. All authors contributed to the article and approved the submitted version.

## Conflict of Interest

The authors declare that the research was conducted in the absence of any commercial or financial relationships that could be construed as a potential conflict of interest.
